# Subjective Outcome Evaluation of the Project P.A.T.H.S.: Findings Based on the Perspective of the Program Implementers

**DOI:** 10.1100/tsw.2007.43

**Published:** 2007-02-09

**Authors:** Daniel T. L. Shek, Andrew M. H. Siu, Tak Yan Lee

**Affiliations:** ^1^ Quality of Life Centre, Hong Kong Institute of Asia-Pacific Studies, Hong Kong; ^2^ Social Welfare Practice and Research Centre, Department of Social Work, The Chinese University of Hong Kong, Shatin, Hong Kong; ^3^ Department of Rehabilitation Sciences, Hong Kong Polytechnic University, Hong Kong; ^4^ Department of Applied Social Studies, City University of Hong Kong, Hong Kong

**Keywords:** positive youth development, Hong Kong, subjective outcome evaluation, worker satisfaction

## Abstract

A total of 52 schools (n = 8679 students) participated in the experimental implementation phase of the Project P.A.T.H.S. (Positive Adolescent Training through Holistic Social Programmes). After completion of the Tier 1 Program, 344 instructors completed the Subjective Outcome Evaluation Form (Form B) to assess their views of the program, instructors, and perceived effectiveness of the program. Based on the consolidated reports submitted by the schools to the funding body, the research team aggregated the consolidated data to form a “reconstructed” overall profile on the perceptions of the program implementers. Results showed that high proportions of the workers had positive perceptions of the program and their own performance, and roughly 90% of the workers regarded the program as helpful to the program participants. The present study provides additional support for the effectiveness of the Tier 1 Program of the P.A.T.H.S. Project in Hong Kong.

### INTRODUCTION

Subjective outcome evaluation is a commonly used strategy to evaluate programs in the context of human services and it has been used by different professionals in different fields, such as education, social work, psychology, medicine, and allied health professions. Although there are many criticisms of this approach, the client satisfaction approach is widely used in different service settings[1,2]. As pointed out by Royse[3], "despite the generally positive bias and the problems associated with collecting representative samples of clients, there is much to recommend client satisfaction studies as one means of evaluating a program. Because professionals do not experience the agency in the same way as the clients, it is important to ask clients to share their experiences" (pp. 264–265).

Although it is important to understand the experiences of the clients, it is equally important to examine the experiences of the workers who conduct the intervention or implement the program. This practice is particularly important for programs that are implemented by workers who are not directly involved in the design of the program. In many positive youth development or adolescent prevention programs, the programs are often developed by academics and experienced workers in the field (e.g., school-based drug prevention programs) with the involvement of some frontline workers. The developed programs are then implemented by frontline workers, such as teachers and social workers. Under such contexts, frontline workers may have strong resistance in the implementation of programs for which they have had little involvement in the design process. Furthermore, organizational constraints may also adversely affect staff morale that, in turn, lowers the motivation of the workers to implement the program in an authentic manner.

There are several reasons why subjective outcome evaluation should include the perceptions of the program implementers. First, since the program implementers are also stakeholders of the developed programs, their views should be gathered. According to the Joint Committee on Standards for Educational Evaluation[4], stakeholders should be identified (Standard U1) and their views should be taken into account (Standard F2). According to utilization-focused evaluation[5], relevant stakeholders should also be involved in the evaluation process. From the interpretive and constructivist perspectives, as the reality is fluid, it is also important to look at the experiences of different stakeholders.

Second, as program implementers are usually more experienced than the clients, it can be argued that their views may be more accurate than those of the clients. For example, in adolescent prevention programs, it is common to ask the program participants and workers about their perceptions of the program design, objectives, and rationales. It can be argued that the program implementers in this context possess better skills and experience in judging the quality of the program designed. Similarly, with their professional training and experience, workers will be in a better position to assess the effectiveness of the programs.

Third, it can be argued that subjective outcome evaluation based on the perspective of the workers is important as far as reflective practice is concerned. According to Osterman and Kottkamp[6], professionals need and desire feedback about their own performance, and personal reflections can lead to professional growth and development. Similarly, Taggart and Wilson[7] pointed out the importance of reflective practice in teaching. As reflective practice has become more important in different disciplines, the practice of subjective outcome evaluation can help professionals to reflect on the program they have delivered, and to assess their input and quality of the implementation. In short, subjective outcome evaluation based on the perspective of the workers constitutes a vehicle that facilitates reflective practice in helping professionals.

Fourth, the inclusion of subjective outcome evaluation based on the worker’s perspective can give the workers a sense of fairness, which is an important determinant of the morale of the workers. Obviously, if only the clients have the right to assess the program implementers, the workers may sense that the evaluation is rather unfair because only the voices of the clients are heard. Furthermore, when the workers are invited to express their views and feelings, they would feel more respected, thus not regarding themselves as the victims of consumerism.

Fifth, in situations where a developed program is used in different sites (e.g., school-based positive youth development programs), implementation experiences may vary across schools. For some sites where the implementation experiences are negative, such news may spread quickly and the related rumors may adversely affect the process of the implementation. As such, if the researchers can build up a systematic profile of the experiences of the workers and disseminate the related findings, such research findings can demystify the rumors and distorted news, and they can help to provide a transparent and accurate picture on the implementation quality.

Finally, based on the principle of triangulation, the collection of subjective outcome evaluation data from different sources definitely can help to answer the question of whether data collected from different sources generate the same picture. For example, while the workers may perceive themselves as performing well in the implementing process, the students may not have the same perceptions. Similarly, the students and instructors may have different views of the learning motivation of the students. In short, inclusion of subjective outcome evaluation data from different perspectives can enable researchers to paint a more complete picture regarding perceived program attributes and effects.

The Project P.A.T.H.S. (Positive Adolescent Training through Holistic Social Programmes) is a two-tier positive youth development program financially supported by the Hong Kong Jockey Club Charities Trust[8,9]. In the Tier 1 Program, a universal positive youth development program typically involving 20-h of training (40 teaching units) in the school year at each grade is provided to Secondary 1 to 3 students at each year. While the experimental implementation phase of the project will last for 3 years (2005–2008), the full implementation phase will last from 2006 to 2009.

Several studies have documented the positive program effects of the Tier 1 Program of the P.A.T.H.S. Project implemented in the experimental implementation phase[10,11,12,13]. With particular reference to subjective outcome evaluation based on students randomly selected from four schools (n = 546 students), results showed that the program participants perceived the program in a favorable manner and roughly 85% of them regarded the program as helpful to them[10]. Interim evaluation based on telephone interviews of the program implementers also showed that nearly all workers (97.1%) regarded the program to be beneficial to the students and most of them (78.6%) had positive global evaluation of the project[13]. While the above studies can give some evidence on the positive aspects of the program, there is a need to know more about the views of the program implementers.

In this paper, subjective outcome evaluation findings based on the perspective of the workers in the Tier 1 Program in the experimental implementation phase are reported. As each participating school was required to submit an evaluation report with the consolidated subjective outcome evaluation profile of the workers to the Hong Kong Jockey Club Charities Trust (i.e., workers were expected to conduct program evaluation as part of their professional practice), we could make use of such reports to “reconstruct” the overall profile of the subjective outcome evaluation data. The major advantage of this strategy is that we can promote practice evaluation in the field while, at the same time, conduct secondary data analyses of the reports submitted.

### METHODS

#### Participants and Procedures

There were 52 schools joining the experimental implementation phase. The mean number of students per school was 166.90 (ranging from 37–240 students), with an average of 4.58 classes per school (ranging from 2–7 classes). Among them, 29 schools adopted the full program (i.e., 20-h program involving 40 units). The mean number of sessions used to implement the program was 17.75 (ranging from 3–50 sessions). While 21 (40.4%) schools incorporated the program in the formal curriculum (e.g., Liberal Studies, Life Education), 31 schools (59.6%) used other modes (e.g., using form master’s periods and other combinations) to implement the program. A total of 419 workers implemented the program in the schools. The mean numbers of social workers and teachers implementing the program per school were 2.63 (ranging from 0–8) and 5.13 (ranging from 0–17), respectively.

After the Tier 1 Program was completed, the workers were invited to respond to a subjective outcome evaluation questionnaire. A total of 344 workers responded to the Subjective Outcome Evaluation Form (Form B) developed by the research team. The data collection was normally carried out after the completion of the program. To facilitate the program evaluation, the research team developed an evaluation manual with standardized instructions for collecting the subjective outcome evaluation data[14]. In addition, adequate training was provided to the workers during the 20-h training workshops on how to collect and analyze the data collected by Form B.

### Instruments

The Subjective Outcome Evaluation Form (Form B) was designed by Daniel Shek and Andrew Siu[14]. Broadly speaking, there are several parts in this evaluation form as follows:

- Program implementer's perceptions of the program, such as program objectives, design, classroom atmosphere, interaction among the students, and the respondent's participation during class (10 items)
- Program implementer's perceptions of his/her own practice, including his/her understanding of the course, teaching skills, professional attitude, involvement, and interaction with the students (10 items)
- The worker's perceptions of the effectiveness of the program, such as promotion of different psychosocial competencies, resilience, and overall personal development (16 items)
- The extent to which the worker would recommend the program to other students with similar needs (1 item)
- The extent to which the worker would teach similar programs in future (1 item)
- Overall satisfaction with the program (1 item)
- Things that the worker obtained from the program (opened-ended question)
- Things that the worker appreciated most (open-ended question)
- Difficulties encountered (open-ended question)
- Areas that require improvement (open-ended question)

The workers collecting the data were requested to input the data in an Excel file developed by the research team, which would automatically compute the frequencies and percentages associated with the different ratings for an item. When the schools submitted the reports, they were also requested to submit the soft copy of the consolidated data sheets. After receiving the consolidated data by the funding body, the data were aggregated to "reconstruct" the overall profile based on the subjective outcome evaluation data.

## RESULTS

The quantitative findings based on the closed-ended questions are presented in this paper. There are several observations that can be highlighted from the findings. First, the participants generally had positive perceptions of the program (see Table 1), including the objectives of the teaching units (90.7%), systematic design of the teaching activities (81.4%), and active involvement of the students (83.4%). Second, a high proportion of the workers had positive evaluation of their performance (see Table 2). For example, 94.1% of the workers had positive evaluation of their performance, 98.5% of the workers expressed that they were concerned about the students, and 97% believed that they had very good professional attitude. Third, as shown in Table 3, many workers perceived that the program promoted the development of students, including their bonding (85.1%), resilience (81.3%), social competence (91.3%), emotional competence (88.3%), moral competence (90.4%), self-understanding (93%), and overall development (89.8%). Fourth, 84.3% of the workers would recommend the program to students with similar needs. Fifth, roughly 85% of the workers expressed that they would teach similar courses again in future. Finally, roughly four-fifths of the respondents indicated that they were satisfied with the program (see Table 4).

### DISCUSSION

In this study, the Subjective Outcome Evaluation findings based on the perspective of the workers showed that a high proportion of the respondents had positive perceptions of the program, including the program design, workers, and effectiveness. The present findings are consistent with the subjective outcome evaluation findings based on Form A (i.e., evaluation based on the students), which also showed that a high proportion of the program participants had favorable perceptions of the program, workers, and helpfulness of the program. Furthermore, the findings are also in line with those evaluation findings based on objective outcome evaluation, process evaluation, and interim evaluation[10,11,12,13]. Generally speaking, the overall picture based on the subjective outcome evaluation findings obtained from different sources is quite positive in nature. From an information flow perspective, the findings can give more transparent information regarding the feelings and experiences of the workers who implement the Tier 1 Program of the P.A.T.H.S. Project.

**TABLE 1**

**Summary of the Views of the Program Implementers**

| | Strongly Disagree (1) | | Disagree (2) | | Slightly Disagree (3) | | Slightly Agree (4) | | Agree (5) | | Strongly Agree (6) | | Participants with Positive Responses (Options 4–6) | |
|---|---|---|---|---|---|---|---|---|---|---|---|---|---|---|
| | N | % | N | % | N | % | N | % | N | % | N | % | N | % |
| The objectives of the curriculum are very clear (n = 344). | 2 | 0.58 | 7 | 2.03 | 23 | 6.69 | 89 | 25.87 | 200 | 58.14 | 23 | 6.69 | 312 | 90.70 |
| The design of the curriculum is very good (n = 344). | 5 | 1.45 | 29 | 8.43 | 58 | 16.86 | 140 | 40.70 | 105 | 30.52 | 7 | 2.03 | 252 | 73.26 |
| The activities were carefully planned (n = 344). | 4 | 1.16 | 16 | 4.65 | 44 | 12.79 | 138 | 40.12 | 131 | 38.08 | 11 | 3.20 | 280 | 81.40 |
| The classroom atmosphere was very pleasant (n = 343). | 0 | 0.00 | 12 | 3.50 | 50 | 14.58 | 144 | 41.98 | 125 | 36.44 | 12 | 3.50 | 281 | 81.92 |
| There was much peer interaction among the students (n = 344). | 1 | 0.29 | 15 | 4.36 | 46 | 13.37 | 149 | 43.31 | 119 | 34.59 | 14 | 4.07 | 282 | 81.98 |
| Students participated actively during lessons (including discussions, sharing, games, etc.) (n = 344). | 1 | 0.29 | 13 | 3.78 | 43 | 12.50 | 167 | 48.55 | 109 | 31.69 | 11 | 3.20 | 287 | 83.43 |
| The program has a strong and sound theoretical support (n = 343). | 2 | 0.58 | 14 | 4.08 | 47 | 13.70 | 126 | 36.73 | 133 | 38.78 | 21 | 6.12 | 280 | 81.63 |
| The teaching experience I encountered enhanced my interest in the course (n = 342). | 8 | 2.34 | 23 | 6.73 | 56 | 16.37 | 115 | 33.63 | 129 | 37.72 | 11 | 3.22 | 255 | 74.56 |
| Overall, I have very positive evaluation of the program (n = 344). | 7 | 2.03 | 28 | 8.14 | 60 | 17.44 | 139 | 40.41 | 105 | 30.52 | 5 | 1.45 | 249 | 72.38 |
| On the whole, students like this curriculum very much (n = 344). | 6 | 1.74 | 29 | 8.43 | 63 | 18.31 | 141 | 40.99 | 100 | 29.07 | 5 | 1.45 | 246 | 71.51 |

In the context of human services, there is a growing emphasis on the importance of understanding the views and experiences of the workers who conduct the intervention. In their discussion of client and worker satisfaction in a child protection agency, Winefield and Barlow[15] argued that monitoring staff perception was important because "staff have valuable first-hand experience of how, when, and how well programs work" (p. 898). With specific reference to school-based prevention programs, Peterson and Esbensen[16] pointed out that "because personnel, consciously or unconsciously, influence the effectiveness of prevention program lessons, it is important to assess their perceptions when evaluating a specific program to provide insight into the context in which the program operates" (p. 219). In the same vein, Flannery and Torquati[17] remarked that "teachers who are not satisfied with a program are less likely to use the program

**TABLE 2**
**Summary of the Views of the Program Implementers about Themselves**

| | Strongly Disagree (1) | | Disagree (2) | | Slightly Disagree (3) | | Slightly Agree (4) | | Agree (5) | | Strongly Agree (6) | | Participants with Positive Responses (Options 4–6) | |
|---|---|---|---|---|---|---|---|---|---|---|---|---|---|---|
| | N | % | N | % | N | % | N | % | N | % | N | % | N | % |
| I have a good mastery of the curriculum (n = 339). | 2 | 0.59 | 11 | 3.24 | 55 | 16.22 | 154 | 45.43 | 114 | 33.63 | 3 | 0.88 | 271 | 79.94 |
| I prepared well for the lessons (n = 330). | 0 | 0.00 | 9 | 2.73 | 42 | 12.73 | 141 | 42.73 | 126 | 38.18 | 12 | 3.64 | 279 | 84.55 |
| My teaching skills were good (n = 327). | 0 | 0.00 | 5 | 1.53 | 45 | 13.76 | 151 | 46.18 | 117 | 35.78 | 9 | 2.75 | 277 | 84.71 |
| I have good professional attitudes (n = 338). | 0 | 0.00 | 2 | 0.59 | 8 | 2.37 | 105 | 31.07 | 197 | 58.28 | 26 | 7.69 | 328 | 97.04 |
| I was very involved (n = 339). | 1 | 0.29 | 2 | 0.59 | 19 | 5.60 | 105 | 30.97 | 179 | 52.80 | 33 | 9.73 | 317 | 93.51 |
| I gained a lot during the course of instruction (n = 329). | 2 | 0.61 | 8 | 2.43 | 53 | 16.11 | 125 | 37.99 | 120 | 36.47 | 21 | 6.38 | 266 | 80.85 |
| I cared for the students (n = 340). | 1 | 0.29 | 1 | 0.29 | 3 | 0.88 | 71 | 20.88 | 206 | 60.59 | 58 | 17.06 | 335 | 98.53 |
| I was ready to offer help to students when needed (n = 339). | 1 | 0.29 | 0 | 0.00 | 3 | 0.88 | 49 | 14.45 | 215 | 63.42 | 71 | 20.94 | 335 | 98.82 |
| I had much interaction with the students (n = 337). | 0 | 0.00 | 6 | 1.78 | 28 | 8.31 | 128 | 37.98 | 150 | 44.51 | 25 | 7.42 | 303 | 89.91 |
| Overall, I have a very positive evaluation of myself as an instructor (n = 340). | 0 | 0.00 | 2 | 0.59 | 18 | 5.29 | 110 | 32.35 | 195 | 57.35 | 15 | 4.41 | 320 | 94.12 |

materials, regardless of whether their principal or district administration is supportive of the program" (p. 395). Unfortunately, there are limited research studies in the literature that document the perceptions of workers in positive youth development and adolescent prevention programs[12]. As pointed out by Najavits et al.[18], there are few empirical studies on therapists' satisfaction with treatment guided by manuals and there is limited understanding of the "inner world of clinicians" (p. 36).

There are three strengths of this study. First, the subjective outcome evaluation findings are based on a large sample size (n = 344 workers involving 52 schools). Such a big sample size substantially enhances the generalizability of the research findings to other student populations. Second, different aspects of subjective outcome including views of the program, workers, perceived effectiveness, and overall satisfaction were covered in the study. Third, the present study demonstrates the strategy of "reconstructing" the overall profile of the subjective outcomes based on the reports submitted by the participating schools. In fact, this study is the first published scientific study utilizing this "reconstruction" approach based on such a large number of workers in the Chinese culture.

On the other hand, it is noteworthy that there are several limitations of the study. First, as the data were reconstructed from the reports submitted by the schools, the unit of analysis was schools rather than individual program participants. As such, characteristics at the individual level cannot be examined. Second, while the reconstructed profile can give some ideas about the global picture, those unfavorable responses were diluted. It is suggested that it is important to examine such unfavorable responses by looking further at the qualitative findings.

**TABLE 3**
**Perceived Effectiveness of the Program by the Program Implementers**

| The extent to which the Tier 1 Program (i.e., the program in which all students have joined) has helped your students (n = No. of responses) | Unhelpful (1) | | Not Very Helpful (2) | | Slightly Helpful (3) | | Helpful (4) | | Very Helpful (5) | | Participants with Positive Responses (Options 3–5) | |
|---|---|---|---|---|---|---|---|---|---|---|---|---|
| | N | % | N | % | N | % | N | % | N | % | N | % |
| It has strengthened students' bonding with teachers, classmates, and their families (n = 343). | 1 | 0.29 | 50 | 14.58 | 178 | 51.90 | 106 | 30.90 | 8 | 2.33 | 292 | 85.13 |
| It has strengthened students' resilience in adverse conditions (n = 343). | 1 | 0.29 | 63 | 18.37 | 179 | 52.19 | 96 | 27.99 | 4 | 1.17 | 279 | 81.34 |
| It has enhanced students' social competence (n = 343). | 1 | 0.29 | 29 | 8.45 | 140 | 40.82 | 152 | 44.31 | 21 | 6.12 | 313 | 91.25 |
| It has improved students' abilities to handle and express emotions (n = 343). | 3 | 0.87 | 37 | 10.79 | 155 | 45.19 | 136 | 39.65 | 12 | 3.50 | 303 | 88.34 |
| It has enhanced students' cognitive competence (n = 343). | 2 | 0.58 | 58 | 16.91 | 176 | 51.31 | 101 | 29.45 | 6 | 1.75 | 283 | 82.51 |
| Students' abilities to resist harmful influences have been improved (n = 343). | 3 | 0.87 | 72 | 20.99 | 164 | 47.81 | 95 | 27.70 | 9 | 2.62 | 268 | 78.13 |
| It has strengthened students' abilities to distinguish between the good and the bad (n = 342). | 0 | 0.00 | 33 | 9.65 | 164 | 47.95 | 132 | 38.60 | 13 | 3.80 | 309 | 90.35 |
| It has increased students' competence in making sensible and wise choices (n = 343). | 2 | 0.58 | 51 | 14.87 | 171 | 49.85 | 110 | 32.07 | 9 | 2.62 | 290 | 84.55 |
| It has helped students to have life reflections (n = 343). | 7 | 2.04 | 67 | 19.53 | 154 | 44.90 | 97 | 28.28 | 18 | 5.25 | 269 | 78.43 |
| It has reinforced students' self-confidence (n = 342). | 2 | 0.58 | 69 | 20.18 | 160 | 46.78 | 99 | 28.95 | 12 | 3.51 | 271 | 79.24 |
| It has increased students' self-awareness (n = 344). | 2 | 0.58 | 22 | 6.40 | 155 | 45.06 | 144 | 41.86 | 21 | 6.10 | 320 | 93.02 |
| It has helped students to face the future with a positive attitude (n = 343). | 4 | 1.17 | 68 | 19.83 | 168 | 48.98 | 95 | 27.70 | 8 | 2.33 | 271 | 79.01 |
| It has helped students to cultivate compassion and care about others (n = 343). | 2 | 0.58 | 73 | 21.28 | 160 | 46.65 | 97 | 28.28 | 11 | 3.21 | 268 | 78.13 |
| It has encouraged students to care about the community (n = 344). | 10 | 2.91 | 83 | 24.13 | 152 | 44.19 | 89 | 25.87 | 10 | 2.91 | 251 | 72.97 |
| It has promoted students' sense of responsibility in serving the society (n = 343). | 11 | 3.21 | 84 | 24.49 | 173 | 50.44 | 67 | 19.53 | 8 | 2.33 | 248 | 72.30 |
| It has enriched the overall development of the students (n = 344). | 1 | 0.29 | 34 | 9.88 | 162 | 47.09 | 131 | 38.08 | 16 | 4.65 | 309 | 89.83 |

The third limitation of the study is that although it is possible to interpret the positive findings in terms of program success, it is noteworthy that there are several alternative explanations of the findings. The first alternative explanation is "beauty on the beholder side" hypothesis. As the workers are the stakeholders and they are personally involved in implementing the program, they tend to look at the program effect and their own performance in a more favorable light. The second alternative explanation is the "cognitive dissonance" hypothesis. As the workers may have beliefs about the value of the program, it would be difficult for them to rate the program and themselves in an unfavorable manner. In particular, unfavorable evaluation would pose a threat to the professional self and self-esteem of the workers. The third alternative explanation is the "survival" hypothesis that maintains that the positive subjective outcome evaluation findings occurred as a result of the participants' anxiety that the program would be cut if the evaluation findings are not positive. This possibility could be partially dismissed because the funding body has never linked funding with program success, and there was no league table in the evaluation findings. The final alternative interpretation is that the workers may consciously respond in a

**TABLE 4**
**Other Aspects of Subjective Outcome Evaluation Based on the Views of the Workers**

**If you have a student whose needs and conditions are similar to those of your students who have joined the program, will you suggest him/her to participate in this program? (n = 338)**

| Definitely Will Not Suggest (1) | | Will Not Suggest (2) | | Will Suggest (3) | | Definitely Will Suggest (4) | | Participants with Positive Responses (Options 3 and 4) | |
|---|---|---|---|---|---|---|---|---|---|
| N | % | N | % | N | % | N | % | N | % |
| 6 | 1.78 | 47 | 13.91 | 236 | 69.82 | 49 | 14.50 | 285 | 84.32 |

**If there is a chance, will you teach similar programs again in the future? (n = 335)**

| Definitely Will Not Teach (1) | | Will Not Teach (2) | | Will Teach (3) | | Definitely Will Teach (4) | | Participants with Positive Responses (Options 3 and 4) | |
|---|---|---|---|---|---|---|---|---|---|
| N | % | N | % | N | % | N | % | N | % |
| 6 | 1.79 | 46 | 13.73 | 232 | 69.25 | 51 | 15.22 | 283 | 84.48 |

**Do you think the implementation of the program has helped you in your professional growth (e.g., enhancement of your skills)? (n = 329)**

| Unhelpful (1) | | Not Very Helpful (2) | | Slightly Helpful (3) | | Helpful (4) | | Very Helpful (5) | | Participants with Positive Responses (Options 3–5) | |
|---|---|---|---|---|---|---|---|---|---|---|---|
| N | % | N | % | N | % | N | % | N | % | N | % |
| 9 | 2.74 | 56 | 17.02 | 142 | 43.16 | 98 | 29.79 | 24 | 7.29 | 264 | 80.24 |

"nice" manner to help the researchers to illustrate positive program effect. However, this alternative explanation could be partially dismissed because negative ratings were recorded (e.g., whether the workers would teach similar courses again) and the workers responded in an anonymous manner. Despite these limitations, the present findings suggest that the Tier 1 Program and its implementation were perceived in a positive manner by the program implementers and the workers perceived the program to be beneficial to the development of the students and the program implementers.

### ACKNOWLEDGMENTS

The preparation for this paper and the Project P.A.T.H.S. were financially supported by the Hong Kong Jockey Club Charities Trust.
